# Incredible affinity of Kattosh with PPAR‐γ receptors attenuates STZ‐induced pancreas and kidney lesions evidenced in chemicobiological interactions

**DOI:** 10.1111/jcmm.17339

**Published:** 2022-05-02

**Authors:** Md. Mamunur Rashid, Md. Atiar Rahman, Md. Shahidul Islam, Md. Amjad Hossen, A. S. M. Ali Reza, A. M. Abu Ahmed, Afnan M. Alnajeebi, Nouf Abubakr Babteen, Mala Khan, Salama Mostafa Aboelenin, Mohamed Mohamed Soliman, Alaa H. Habib, Hend F. Alharbi

**Affiliations:** ^1^ Department of Biochemistry and Molecular Biology University of Chittagong Chittagong Bangladesh; ^2^ 118862 Department of Pharmacy Faculty of Science and Engineering International Islamic University Chittagong Chittagong Bangladesh; ^3^ Department of Genetic Engineering and Biotechnology University of Chittagong Chittagong Bangladesh; ^4^ 441424 Department of Biochemistry College of Science University of Jeddah Jeddah Saudi Arabia; ^5^ Bangladesh Reference Institute for Chemical Measurements (BRiCM) Dhaka Bangladesh; ^6^ 125895 Biology Department Turabah University College Taif University Taif Saudi Arabia; ^7^ 125895 Clinical Laboratory Sciences Department Turabah University College Taif University Taif Saudi Arabia; ^8^ 37848 Department of Physiology College of Medicine King Abdulaziz University Jeddah Saudi Arabia; ^9^ 89660 Department of Food Science and Human Nutrition College of Agriculture and Veterinary Medicine Qassim University Buraidah Saudi Arabia

**Keywords:** diabetes mellitus, ethyl alpha‐D‐glucopyranoside, histopathological examination, *Lasia spinosa*, phytochemical screening, α‐amylase

## Abstract

Since ancient times, plants have been used as green bioresources to ensure a healthier life by recovering from different diseases. Kattosh (*Lasia spinosa* L. Thwaites) is a local plant with various traditional uses, especially for arthritis, constipation and coughs. This research investigated the effect of Kattosh stem extract (LSES) on streptozotocin‐induced damage to the pancreas, kidney, and liver using in vitro, in vivo and in silico methods. In vitro phytochemical, antioxidative and anti‐inflammatory effects of LSES were accomplished by established methods followed by antidiabetic actions in in vivo randomized controlled intervention in STZ‐induced animal models for four weeks. In an in silico study, LSES phytocompounds interacted with antidiabetic receptors of peroxisome proliferator‐activated receptor‐gamma (PPAR, PDB ID: 3G9E), AMP‐activated protein kinase (AMPK, PDB ID: 4CFH) and α‐amylase enzyme (PDB ID: 1PPI) to verify the in vivo results. In addition, LSES showed promising in vitro antioxidative and anti‐inflammatory effects. In contrast, it showed a decrease in weekly blood glucose level, normalized lipid profile, ameliorated liver and cardiac markers, managed serum AST and ALT levels, and increased glucose tolerance ability in the animal model study. Restoration of pancreatic and kidney damage was reflected by improving histopathological images. In ligand–receptor interaction, ethyl α‐d‐glucopyranoside of Kattosh showed the highest affinity for the α‐amylase enzyme, PPAR, and AMPK receptors. Results demonstrate that the affinity of Kattosh phytocompounds potentially attenuates pancreatic and kidney lesions and could be approached as an alternative antidiabetic source with further clarification.

## 1 INTRODUCTION

Diabetic mellitus (DM) is a metabolic condition that affects millions of people worldwide. It is caused by a shortage of insulin or inadequate insulin synthesis in the pancreas, leading to severe complications such as diabetic neuropathy, nephropathy, retinopathy and cardiovascular disease.^1^ According to a recent study of diabetes prevalence worldwide, the disease now affects 463 million people.^2^

Despite the availability of various natural and synthetic antidiabetic drugs, diabetes and its micro‐ and macroimplications are a severe medical concern globally. Modern diabetic treatments are typically connected with problems such as ineffectiveness, high cost and a wide range of side effects.^3^ Side effects of antidiabetic sulfonylureas include heartburn, vomiting and skin rashes. If taken for a long time, biguanides (metformin) might cause gastrointestinal problems, anorexia, vomiting and B12 deficiency.^4^ Because of the drawbacks of conventional medicines, medicinal plants with claimed antidiabetic activity may be used as an alternative source for the management of diabetes, especially in developing countries, because of their easy accessibility, cost‐effectiveness, cultural acceptability and lower side effects.^5^ A list of 21,000 medicinal plants from throughout the world has been compiled by the World Health Organization (WHO). There are about 400 plants that can be used to cure diabetes.^6^ Kattosh is one such plant that has been utilized to treat several ailments by traditional healers in Bangladesh and other areas of the world.

*Lasia spinosa*, also known as Kattosh or Kantakachu belonging to the Araceae family, is a perennial herbaceous plant that grows 1 to 2 metres tall and spreads via a long, creeping stoloniferous stem. It is primarily found in Tropical Asia, from India to New Guinea and South‐East Asia, including Bangladesh. Since time immemorial, all parts of this plant have been used for treating a wide range of ailments and diseases in several Asian countries.^7^ The stem is used as an expectorant and antitussive in baths to relieve itching caused by roseola, measles, rubella, and other skin illnesses. The tubers are used to cure constipation, rheumatoid arthritis, hyperglycaemia and blood purification in Bangladesh's Rajshahi and Natore regions.^8^ The Naga tribes of India believe that the leaves of LS have anthelminthic effects, and they have used them to treat parasitic intestinal worms since ancient times.^9^ However, no comprehensive work on the antidiabetic effects of *Lasia spinosa* is yet conducted by any researcher. Therefore, the current research aimed to explore the potential effect of Kattosh (ethanol extract of *Lasia spinosa* stem, LSES) to recover the lesions of the pancreas, liver and kidney in the streptozotocin‐induced albino rat model.

## 2 MATERIALS AND METHODS

### 2.1 Chemicals

Ethanol, starch, iodine, α‐amylase, potassium monohydrogen phosphate, potassium dihydrogen phosphate, potassium trihydrogen phosphate, potassium trihydrogen phosphate, potassium trihydrogen phosphate acarbose, Tris–HCL buffer, hydrochloric acid, thiobarbituric acid (TBA), trichloroacetic acid (TCA), butylated hydroxytoluene (BHT), potassium chloride, ferric chloride, sodium chloride, dextrose, sodium citrate, sodium phosphate buffer, BSA, aspirin, phosphate buffer, citric acid, streptozotocin (STZ), picric acid, formalin (100%), anthrone, ethanol (98%), glucose, xylene, wax/paraffin, glycerine, Mayer’s fixative, iron haematoxylin, eosin and DPX were supplied by Sigma‐Aldrich (USA). Food grade sugar and D‐fructose were purchased from local suppliers. Unless otherwise noted, all reagents and chemicals were guaranteed analytical grade.

### 2.2 Plant material collection and extraction

Kattosh stems were collected from Fatehabad, an area under Chittagong district, Bangladesh. Dr. Sheikh Bokhtear Uddin, Taxonomist and Professor at the University of Chittagong’s Department of Botany, identified the plant. For future reference, a plant sample was preserved with the accession number LAMLS‐A120.

### 2.3 Extract preparation

The plant stem was washed and cleaned before being sliced and dried at room temperature in the shade. It was dried overnight at 40°C in the oven before grinding and then ground using a mechanical grinder. The resultant powder (800 g) was steeped in 2.0 L of ethanol at room temperature (23 ± 0.5°C) for 7 days, with 2 to 3 days of intervals, with occasional stirring. A rotary evaporator (Bibby Scientific, RE200, UK) was used to condense the collected supernatant under reduced pressure at a temperature below 50°C. The concentrated crude extract (LSES) was kept in a Petri dish and dried at 37°C for complete evaporation of the solvent. For future use, the concentrated dark brown semisolid ethanolic stem extract (LSES, 8.0 g) was kept at 4°C.

### 2.4 Phytochemical group test (qualitative)

The fractions were submitted to qualitative screening for phytochemical groups using a well‐established method. Alkaloids, glycosides, steroids, flavonoids, tannins, saponins, phlobatannins, carbohydrates and proteins were all measured in a 10% (w/v) extract solution for each test.^9^

### 2.5 Gas chromatography–mass spectroscopy analysis (GC‐MS)

The bioactive compounds extracted from the LSES were analysed by gas chromatography (GC‐2010 Plus; Shimadzu Corporation, Kyoto, Japan), coupled with a mass spectrometer (GC‐MS‐TQ 8040; Shimadzu Corporation, Kyoto, Japan). A fused silica capillary column (Rxi‐5ms; 30 m, 0.25 mm ID and 0.25 μm) was used for GC maintaining sample inlet temperature at 250°C. 1 µl sample was injected in *split‐less* mode. The oven temperature was programmed as 75°C (1 min); 25°C, 125°C (1 min); and 10°C, 300°C (15 min). The aux (GC‐to‐MS interface) temperature was set to 250°C. The total run time was 36.50 min, and the column flow rate was 1.5 ml/min He gas. An electron ionization (EI)‐type mass spectroscopy (MS) was used in a Q3 scan mode. 200°C ion source temperature, 250°C interface temperature, 1.17 kV detector voltage and 50–1000 m/z mass range were set for MS. Individual compound with m/z ratio was searched in ‘NIST‐MS Library 2014’. The total ionic chromatogram (TIC) was used to determine the peak area and the percentage amounts of each compound.

### 2.6 Quantitative phytochemical analysis

#### 2.6.1 Determination of total phenolic content (TPC)

The total phenolic content of LSES was determined using a technique described by Singleton and Rossi (1965) with minor modifications.^10^ In brief, 5 ml LSES (8 mg/10 ml) or standard (gallic acid) was added to test tubes, followed by a 2.5 ml Folin‐Ciocalteu reagent. After that, 2.5 ml of 7.5% Na2CO_3_ was added to the test tubes. Then, the test tubes of all tests were incubated at 25°C for 20 min. Finally, the optical density (OD) of all the test tubes was measured at 760 nm using a UV‐VIS 1280 spectrophotometer (Shimadzu Corporation, Japan). All concentrations were tested in triplicates, with distilled water serving as a blank.

TPC was calculated using the following equation:

$$C=\frac{\left(c\times V\right)}{m};$$

where ‘C’ is the TPC (mg/g plant extract in GAE), ‘c’ is the sample concentration obtained from the calibration curve (mg/ml), ‘V’ is the sample volume, and m is the sample weight (g). The total phenolic content of LSES was determined as mean ± SD and expressed as gallic acid equivalents (GAE) per gram of plant extract.

#### 2.6.2 Determination of total flavonoid content (TFC)

The total flavonoid content of LSES was determined using Kumaran and Karunakaran's conventional technique, with certain modifications.^11^ Initially, 1 ml of LSES (8 mg/10 ml) or 1 ml of standard solution was put in test tubes. The test tubes were then filled with 3 ml of ethanol. After that, 200 L of 10% AlCl_3_ was added to all the test tubes holding 200 L of 1 M CH_3_COOK. The test tubes were then filled with 5.6 ml of distilled water and incubated at 30 min. Finally, a spectrophotometer was used to detect absorbance at 415 nm. All experiments were done in triplicate, using water as a control:

$$C=\frac{\left(c\times V\right)}{m}$$

where ‘C’ is the TFC (mg/g LSES in Rutin), ‘c’ is the sample concentration obtained from the calibration curve (mg/ml), ‘V’ is the sample volume, and m is the sample weight (g). The experiment was repeated three times, with the results presented as mean ± SD, and values were expressed in mg of Rutin equivalent (RE)/g of dried extracts.

#### 2.6.3 Determination of total antioxidant capacity (TAC)

The phospho‐molybdenum method was used to assess the total antioxidant capacity of LSES^12^. To begin, 0.5 ml of LSES or standard was added to 3 ml of the reaction mixture, including 1 ml of 1.8 M H_2_SO_4_, 1 ml of.084 M dipotassium hydrogen phosphate and 1 ml of % ammonium molybdate. The test tubes were then heated to 95°C for 10 min before cooling to room temperature for another 10 min. A spectrophotometer was used to test the final solution's absorbance at 695 nm. The experiment was repeated three times.

The following equation was used to compute total antioxidant capacity,

$$C=\frac{\left(c\times V\right)}{m};$$

where ‘C’ stands for total antioxidant capacity; ‘c’, for sample concentration (mg/ml); ‘V’, for sample volume; and ‘m’, for sample weight. The results were quantified as mg of ascorbic acid equivalent per gram of dry extracts.

#### 2.6.4 Determination of total proanthocyanidin content (TPACC)

With slight modification, Broadhurst's method was employed to determine proanthocyanidins, with catechin as a reference component.^13^ In test tubes, 300 ml of LSES or standard was mixed with 1.8 ml of a 4 per cent w/v vanillin–ethanol solution. The reaction mixture was then combined with 900 µl of a 4 per cent v/v hydrochloric acid–ethanol solution and kept at room temperature for 15 min. Finally, the absorbance of the reaction mixture was measured at 500 nm using a spectrophotometer. The total proanthocyanidin content was calculated using the equation below

$$C=\frac{\left(c\times V\right)}{m},$$

where ‘C’ is the total proanthocyanidin content (mg/g LSES in catechin), ‘c’ is the sample concentration determined from the calibration curve (mg/ml), ‘V’ is the sample volume, and m is the sample weight. The results were given in milligrams of catechin equivalent per gram of dried extracts.

#### 2.6.5 Free radical scavenging action of DPPH

The antioxidant activity of LSES was compared with that of the conventional antioxidant ascorbic acid. Blois's (1958) methodologies were used to evaluate the complete procedure, with some modifications.^14^ Briefly, ascorbic acid and LSES were diluted to 800, 400, 200, 100 and 50 µg/ml. Then, 0.158 g of DPPH was dissolved in 100 ml of ethanol and maintained in a conical flask with aluminium foil covering it to protect it from light. Next, 2 ml of various doses of LSES and standard was combined with 3 ml of DPPH solution in test tubes. After that, the reaction mixture was incubated in the dark for 30 min at room temperature. Finally, the absorbance of the reaction mixture was measured at 517 nm using a visible spectrophotometer. The control was made the same way as the sample but without the sample. The following formula was used to compute the percentage of DPPH free radical scavenging activity:

$$\text{Percentage}\;\text{of}\;\text{DPPH}\;\text{free}\;\text{radical}\;\text{scavenging}\;\text{activity}=\left\{\frac{\left(\text{Control}\;\text{Absorbance}‐\text{Sample}\;\text{Absorbance}\right)}{\text{Control}\;\text{Absorbance}}\right\}\times 100$$

The percentage of DPPH free radical scavenging activity or inhibition was plotted against concentration, and the IC_50_ (the sample concentration necessary to scavenge 50% of DPPH) was estimated using linear regression analysis.

#### 2.6.6 Measuring of iron‐chelating activity

The effect of LSES on iron chelation was studied compared with the common antioxidant ascorbic acid. The entire technique was carried out with minor adjustments according to Benzie and Strain's established procedure.^15^ In a nutshell, 2 ml of LSES or standard (ascorbic acid) in various concentrations (50–800 µg/ml) was mixed with 1 ml of ethanol and 1, 10‐phenanthroline. The reaction mixture was then incubated at 25°C for 10 min with 2 ml of 200 M FeCl_3_ solution added to all test tubes. Finally, a spectrophotometer was used to measure optical density (OD) at 510 nm. The control was made in the same way as the sample but without the sample or the standard. For three times, the operation was repeated. The iron‐chelating activity was calculated using the equation below:

$$\begin{array}{c} \text{Percentage}\;\text{of}\;\text{iron chelating}\;\text{activity}=\left\{\frac{\left(A_{\text{test}}‐A_{\text{control}}\right)}{A_{\text{control}}}\right\}\times 100 \end{array}$$

where A_test_, absorbance of extract/Standard solution and A_1_, absorbance of control.

The IC_50_ (the sample concentration necessary to scavenge fifty per cent of the iron‐chelating agent) was computed from the plot of percentage of inhibition against extract concentration using linear regression analysis.

#### 2.6.7 Measuring the activity of nitric oxide scavenging

The LSES nitric oxide scavenging activity was measured by comparing it with a standard (quercetin) and modifying Marcocci's approach.^16^ Briefly, 1 ml of LSES or standard solution (18.25, 37.50, 75, 150 and 300 µg/ml) was mixed with 1 ml sodium nitroprusside (5 mM in 20 mM phosphate buffer, pH 7.4) and incubated at 25°C for 150 min. After the incubation period, all the test tubes were given 2 ml of Griess reagent (equal volume of 0.2 per cent w/v sulphanilamide in 5% phosphoric acid and 0.2 per cent w/v naphthylenediamine dihydrochloride). Finally, a spectrophotometer was used to detect absorbance at 546 nm. A similar excluded sample was used for the control. The following formula was used to compute nitric oxide scavenging activity:

$$\text{Nitric}\;\text{oxide}\;\text{scavenging}\;\text{activity}=\left\{\frac{\left(A_{0}‐A_{1}\right)}{A_{0}}\right\}\times 100.$$

A_0_ denotes the absorbance of the control, while A_1_ denotes the absorbance of the extract/standard. Using linear regression analysis, the fraction of scavenging activity or inhibition was plotted versus concentration, and the graph was used to establish the IC_50_.

#### 2.6.8 Hydroxyl radical scavenging activity

The hydroxyl radical scavenging activity of LSES was determined by the method described by Ali Reza.^17^ The hydroxyl radical scavenging activity in an aqueous media was measured using deoxyribose. The generation of malondialdehyde chromogen owing to 2‐deoxyribose breakdown was used to determine hydroxyl radicals. The assay mixture contained 100 ml of 28 mM 2‐deoxyribose dissolved in phosphate buffer, pH 7.4, 500 ml of various concentrations of LSES in buffer, 200 ml of 200 mM ferric chloride (1:1 v/v) and 1.04 mM EDTA, 100 ml of 1 mM hydrogen peroxide, and 100 ml of 1 mM hydrogen peroxide in a final volume of 1 ml. The degree of free radical damage imposed on the substrate deoxyribose was determined using the thiobarbituric acid (TBA) assay after incubation of the test sample at 37°C for 1 h. Deoxyribose degradation inhibition was estimated as a percentage.

$$\text{Hydroxyl}\;\text{radical}\;\text{scavenging}\;\text{activity}=\left\{\left(\frac{A_{0}‐A_{1}}{A_{0}}\right)\times 100\right\}$$

where A_0_ is the absorbance of the control, A_1_ is the absorbance of the extract samples, and A2 is the absorbance of the reference. The IC_50_ value was determined by plotting the percentage of inhibition against the extract concentration.

#### 2.6.9 Lipid peroxidation inhibitory effect

The lipid peroxidation inhibition assay of LSES was carried out using Ruberto's method.^18^ To begin, a test tube was filled with 100 µl of LSES or standard, 500 µl of bovine brain homogenate solution and 200 l of 0.2 mM ferric chloride. The reaction mixture was then incubated for 30 min at 37°C before adding 2 ml of 1% ice‐cooled TBA‐TCA‐BHT solution. The reaction mixture was then placed on ice after 60 min of incubation at 90°C. The reaction mixtures were then centrifuged for 10 min at 56 *g*, and the supernatants were collected to determine the absorbance at 532 nm with a spectrophotometer. The following formula was used to calculate lipid peroxidation activity:

$$\text{Lipid}\;\text{peroxidation}\;\text{activity}=\left\{\left(\frac{\text{Control}\;\text{Absorbance}‐\text{Sample}}{\text{Standard}\;\text{Absorbance}}\right)\right\}\times 100.$$

The IC50 value (the sample concentration necessary to scavenge 50% of lipid peroxidation) was determined by plotting the percentage of inhibition against the extract concentration, and the findings were expressed as mean ± SD.

#### 2.6.10 Membrane stabilization activity

The membrane‐stabilizing activity assay was carried out using a well‐established standard method.^19^ First, four millilitres of fresh blood was drawn from subjects. The Alsever solution (4 ml) was then added. The solutions were centrifuged for 10 min at 3000 *g*. The packed cells were then washed with isosaline. After making a 10% v/v suspension solution, it was stored in the freezer at 4°C. Then, 0.5 mg/ml plant extract or standard was mixed in 10 ml distilled water to make a stock solution with a 500 µg/ml final concentration. The 250, 125, 61.5 and 31.25 mg/ml concentrations were achieved through serial dilution. Next, samples, standards and solvents (1 ml) of various concentrations were put in test tubes (triplicate) with 0.5 ml RBC suspension. Then, 2 ml of hypotonic solution was dissolved (10 Mm). At 37°C, the reaction mixtures were incubated for 30 min. After incubation, the solutions were centrifuged for 20 min at 3000 *g*. After then, the supernatant was collected, and the absorbance at 560 nm was measured. Except for the samples, all reagents were used to make the control. Membrane stabilization activity was calculated using the following formula:

$$\text{Membrane}\;\text{stabilization}\;\text{activity}=\left\{\left(\frac{\text{Control}\;\text{Absorbance}‐\text{Sample}}{\text{Standard}\;\text{Absorbance}}\right)\right\}\times 100.$$

The IC_50_ value (the sample concentration needed to scavenge 50% of membrane stabilization) was determined by plotting the percentage of inhibition against the extract concentration, and the findings were represented as mean ± SD.

#### 2.6.11 Protein denaturation Inhibition

Williams previously reported the anti‐inflammatory effects of crude and fractionated plant extracts using a modified version of the BSA test.^20^ First, 0.5 mg/ml plant extract or standard (aspirin) was diluted in 10 ml distilled water to make a stock solution with a 500 µg/ml final concentration. The 250, 125, 61.5 and 31.25 mg/ml concentrations were achieved through serial dilution. Next, 0.05 ml extract or aspirin and 0.45 ml BSA were added to the reaction mixture in test tubes (triplicate) at different concentrations. The pH of each solution was adjusted to 6.3 using 1 N HCl. After incubation at 37°C for 20 min, the mixtures were heated to 57°C for 30 min.

$$\text{Protein}\;\text{denaturation}\;\text{activity}=\left\{\left(\frac{\text{Control}\;\text{Absorbance}‐\text{Sample}}{\text{Standard}\;\text{Absorbance}}\right)\right\}\times 100.$$

Plotting the percentage of inhibition versus the extract concentration yielded the IC_50_ value (the sample concentration necessary to scavenge 50% of protein denaturation).

#### 2.6.12 In vitro α‐amylase inhibition assay

The inhibitory action of α‐amylase was tested using the starch‐iodine approach^21^ (Hossain et al., 2009). First, a 20 ml of α‐amylase solution (10 mg/ml) mixed in phosphate buffer (0.02 M, pH 7.0, with sodium chloride 0.006 M) was added to test tubes (triplicate) and incubated for 10 min at 37°C. Following incubation, 200 µl of 1% starch solution was added to each test tube, and the mixtures were reincubated at 37°C for 1 h before adding 200 µl of 1% iodine solution and 10 ml of distilled water to each test tube. Finally, a spectrophotometer was used to measure the absorbance of the reaction mixtures at 565 nm. The same conditions were used for the sample, substrate and α‐amylase blank.

### 2.7 In vivo experimental studies

#### 2.7.1 Experimental animals and ethical statements

For the investigation, male adult albino rats of the Wistar strain (Avg. 150–200 g body weight) were employed. They were taken from Jahangirnagar University's animal breeding unit in Dhaka. In a 12‐h light–dark cycle, all the animals were separately housed in polycarbonate cages filled with wood husk at a temperature of (25 ± 2°C) and humidity of 55%–60%. All the rats were fed a commercial rat pellet diet throughout the trial. All targeted animal studies adhered to the Animal Ethics Review Board's rules and regulations at the University of Chittagong (approval no. EACUBS2018‐5).

#### 2.7.2 Acute toxicity evaluation

The acute toxicity test was conducted in a traditional laboratory setting, in accordance with the ‘Organization for Environmental Control Development’ criteria (OECD: Guidelines 420; Fixed‐Dose Method). Each of the allocated animals (*n* = 6) received a single oral dose of LSES (500–2000 mg/kg). The rats fasted overnight before receiving the extract, and their meals were postponed for 3 to 4 h. Following administration, food was withheld for 3–4 h. Individual animals were observed for the first 30 min following dosing, then every 24 min for the next 24 h (with special attention for the first 4 h), with particular attention paid to any unusual reactions, such as changes in the skin, fur, eyes, mucous membranes, respiratory system, autonomic system and central nervous systems, behavioural pattern and allergic syndromes. As an effective therapeutic dose, the median fatal dose (LD_50_ > 2.0 g/kg) was employed.^22^

#### 2.7.3 Diabetes induction

After acclimation, overnight fasted Wistar albino male rats were given 50 mg/kg of streptozotocin (STZ) intraperitoneally according to Mostafavinia.^23^ STZ was dissolved in a citrate buffer with a pH of 0.1 M and a pH of 4.5. In the typical control group, just citrate buffer was administered at the same volume as STZ. All the animals were provided free access to water and pellet food after 30 min of STZ treatment. The animals were kept under constant observation, and a mini‐portable glucometer was used to measure the fasting blood glucose level seven days following STZ injection (Accu‐Chek, Japan). Diabetic rats were recruited for the tests because their fasting blood glucose levels were 16.66 millimoles per litre (mmol/L). The animals were given therapy, and their body weight, blood glucose level, food and hydration intake were all kept track of.

#### 2.7.4 Grouping and dosing of animals

Animals were divided into 6 groups of six rats each after fasting blood glucose levels were measured. Groups were denoted by NC, DC, RC, LSES50, LSES100 and LSES200, where NC, normal control (normal rat without diabetes that was treated with only citric buffer); DC, diabetic control (diabetic rat receiving no treatment); RC, reference control where metformin (Brand Name: Comet from Square Pharmaceuticals Ltd., Bangladesh) is used at 125 mg/kg; LSES50, diabetic rats treated with 50 mg/kg of ethanol stem extract; LSES100, diabetic rats treated with 100 mg/kg of ethanol stem extract; and LSES200, diabetic rats treated with 200 mg/kg of ethanol stem extract. Every week (total 4 weeks), each fasting blood glucose level and body weight was measured and data were recorded. Food and fluid intake was also observed.

#### 2.7.5 Test for oral glucose tolerance (OGTT)

At the end of the third week, each animal's glucose tolerance capacity was assessed using the oral glucose tolerance test (OGTT). Blood samples were taken from the tail tip incision at 0 (immediately before glucose ingestion), 30 to 120 min after glucose delivery. The glucose levels were taken on a regular basis.^24^

#### 2.7.6 Collection of animal organs and blood

At the end of the intervention, the animals were sacrificed using halothane anaesthesia, and blood and organs were collected. A heparinized tube was used to collect whole blood from a heart puncture. The obtained blood was centrifuged for 15 min at 3000 rpm at 25–37°C, and the serum was kept at −20°C for analysis. To estimate hepatic glycogen, each animal's pancreas, kidney and liver were removed, and the liver was cleansed with 0.9% NaCl (normal saline), wiped with tissue paper, weighed and stored at 40°C. For histological evaluation, the pancreas and kidneys were weighed and preserved in plastic vials with 10% buffered formalin.^25^

#### 2.7.7 Analytical methods

Total cholesterol (TC), triglyceride (TG), serum creatinine (SC), serum uric acid (SUA), serum urea (SU), creatine kinase‐MB (CK‐MB), lactate dehydrogenase (LDH), high‐density lipoprotein (HDL) and low‐density lipoprotein (LDL) were measured using reaction kits on a semi‐autoanalyser (Humalyzer 3000, Human). The phenol sulfuric acid method, described by Lo et al., was used to estimate the amounts of liver glycogen.^26^

#### 2.7.8 Histopathological assay

The effect of LSES on streptozotocin‐induced diabetes was studied using histopathological assays of the pancreas and kidney. The preserved tissues were dehydrated with ethanol and cleaned with xylene before being embedded in paraffin wax and cut into 3‐ to 5‐m‐thick sections for microscopic inspection and the creation of histopathology slides. Under the Olympus BX51 Microscope, different kidney and pancreatic cellular condition parameters were evaluated, and histopathology pictures were acquired with the Olympus DP20 system. Mawa et al. (2019) revealed how histological alterations in the pancreatic and kidney were assessed.^27^

### 2.8 Statistical analysis

The data are presented as a mean with a standard deviation. The data were examined using one‐way ANOVA and Tukey's multiple range post hoc tests using statistical software (Statistical Package for the Social Sciences, SPSS Version 22.0; IBM Corporation, NY). *p* > 0.05 was used to determine whether the values were significantly different.

### 2.9 In silico study

#### 2.9.1 Computational studies

##### Molecular docking analysis

The receptors/enzymes for molecular docking were chosen based on a literature review of antidiabetic efficacy. The crystal structure of the following proteins PPAR‐γ (PDB ID 3G9E), AMPK (PDB ID: 4CFH) and α‐amylase enzyme (PDB ID: 1PPI) for the antidiabetic activity was imported from the Protein Data Bank (PDB), an online database (https://www.rcsb.org/), and best binding sites were selected using an online tool Pock Drug.^28^ Besides, the chemical structure of major identified compounds from LSES by GC‐MS was extracted from the PubChem repository (https:/pubchem.ncbi.nlm.nih.gov/). The molecular docking study is briefly described in Hossen et al.^29^

#### 2.9.2 Evaluation of pharmacokinetic parameters

Lipinski's rule of five and Veber's rules were used to analyse the absorption, distribution, metabolism, excretion and toxicity (ADME/T) properties of LSES (number of rotatable bonds; topological polar surface area). SwissADME (http://www.swissadme.ch/) assessed the ADME/T property analysis.^30^

## 3 RESULTS

### 3.1 Qualitative screening for phytochemicals

The qualitative phytochemical analysis of secondary metabolites in the LSES was carried out. Where alkaloids, flavonoids, steroids, tannins, carbohydrates and protein were found, no saponins, phlobatannins or cardiac glycosides were present in the LSES (Table S1).

### 3.2 Gas chromatography–mass spectroscopy (GC‐MS) data for LSES

The GC‐MS analysis of LSES yielded 34 compounds with retention times ranging from 10.117 to 30.261, as indicated in Table 1, and the chromatogram is shown in Figure S1. Among the 34 compounds, the following bioactive metabolites with their large peak area are enlisted as follows: ethyl alpha‐d‐glucopyranoside (PubChem ID, 14811165), hexadecanoic acid, methyl ester (PubChem ID, 1160864), 2‐hydroxy‐1‐(hydroxymethyl)ethyl ester (PubChem ID, 1423165), hexadecanoic acid, 13‐docosenamide, (Z)‐(PubChem ID, 114414729) and nonadecanamide (PubChem ID, 114414729).

**TABLE 1:** Compounds identified in the LSES by GC‐MS

S. N	Compound names	Molecular formula	Molecular weight (g/mol)	Running time	Area
1	Ethyl alpha‐d‐glucopyranoside	C_8_H_16_O_6_	208.21	10.117	14,811,165
2	[3‐(2,3‐Epoxypropoxy)‐propyl]‐trimethoxysilane	C₉H₂₀O₅Si	236.34	10.832	33,099
3	Beta‐D‐ribopyranoside, methyl 2,3,4‐tri‐O‐methyl‐ribopyranoside	C_9_H_18_0_5_	206.24	11.752	57,307
4	Hexadecanoic acid, methyl ester	C_17_H_34_O_2_	270.45	13.501	1,160,864
5	Hexadecanoic acid, ethyl ester	C_18_H_36_O_2_	284.47	14.204	804,775
6	9,12‐Octadecadienoic acid, methyl ester, (E,E)‐	C_19_H_34_O_2_	294.47	15.282	30,865
7	Methyl stearate	C_19_H_38_O_2_	298.50	15.531	544,292
8	E,E‐1,9,17‐docasatriene	C_22_H_40_	304.60	15.282	30,865
9	Cyclopropaneoctanoic acid, 2‐[[2‐[(2‐ethylcyclopropyl) methyl] cyclopropyl] methyl]‐, methyl ester	C_22_H_38_0_2_	334.54	15.867	91,840
10	7‐Tetradecenal, (Z)‐	C_14_H_26_O	210.35	16.301	20,663
11	Hexadecanoic acid, ethyl ester	C_18_H_36_O_2_	284.47	16.168	111,724
12	Cyclopropanetetradecanoic acid, 2‐octyl‐, methyl ester	C_26_H_50_O_2_	394.67	16.258	81,677
13	Dodecanoic acid, 3‐hydroxy‐,ethyl ester	C_14_H_28_O_3_	244.37	17.424	24,934
14	Hexadecanoic acid, 1‐(hydroxymethyl)‐1,2‐ ethanediyl ester	C_35_H_68_O_5_	568.91	17.251	25,653
15	Cyclopropanetetradecanoic acid, 2‐octyl‐, methyl ester	C_26_H_50_O_2_	394.67	17.700	72,655
16	3‐Trifluoroacetoxypentadecane	C_17_H_31_F_3_O_2_	324.42	19.448	21,160
17	Cyclopentadecanone, oxime	C_15_H_29_NO	239.40	19.539	109,615
18	Hexanoic acid, octadecyl ester	C_24_H_48_O_2_	368.63	19.883	187,501
19	Hexadecanoic acid, 2‐hydroxy‐1‐(hydroxymethyl) ethyl ester	C_19_H_38_O_4_	330.50	20.231	1,423,165
20	Bis(tridecyl) phthalate	C_34_H_58_O_4_	530.82	20.607	292,431
21	Cyclopentadecanone, oxime	C_15_H_29_NO	239.40	22.053	89,043
22	9,9‐Dimethoxybicyclo[3.3.1]nona‐2,4‐dione	C_11_H_16_O_4_	212.24	23.511	75,091
23	2,3‐Dihydroxypropyl icosanoate, 2TMS derivative	C_29_H_62_O_4_Si_2_	530.97	23.513	180,350
24	Octadecanoic acid, 2,3‐dihydroxypropyl ester	C_21_H_42_O_4_	358.55	23.869	774,801
25	13‐Docosenamide, (Z)‐	C_22_H_43_NO	337.58	24.611	11,441,472
26	Nonadecanamide	C_19_H_39_	297.51	24.611	11,441,472
27	Cyclononasiloxane, octadecamethyl‐	C_18_H_54_O_9_Si_9_	667.38	25.471	175,705
28	Cyclodecasiloxane, eicosamethyl‐	C_20_H_60_O_10_Si_10_	741.53	28.097	93,043
29	Acetoxyacetic acid, 2‐(1‐adamantyl)ethyl ester	C_16_H_24_O_4_	280.36	28.490	2,717,052
30	Phenol, 4‐(1,1,3,3‐tetramethylbutyl)‐	C_14_H_22_O	206.32	28.490	2,717,052
31	Dihydroartemisinin, 10‐O‐(t‐butyloxy)‐	C_19_H_32_0_6_	356.50	30.261	71,783
32	Stigmasterol	C_29_H_48_O	412.70	30.261	75,044
33	3.beta.‐Stigmast‐5‐en‐3ol, flophemesyl ether	C_37_H_55_F_5_OSi	638.90	30.261	75,044
34	Ergost‐5,8(14)‐dien‐3‐ol	C_28_H_46_O	398.70	30.261	75,044

### 3.3 Antioxidant potentiality of LSES

#### 3.3.1 Total phenolic content (TPC), total flavonoid content (TFC), total antioxidant capacity (TAC), total proanthocyanidin content (TPACC) and antioxidative capacities of LSES

Table 2 summarizes the TPC, TFC, TAC and TPACC of LSES. The gallic acid equivalents (GAE) per gram of plant extract were used to calculate the TPC of LSES. The phenolic content in LSES was found to be precisely 130.72 ± 1.09 mg/g dry weight. The Rutin equivalent per gram of LSES was used to calculate TFC. Using the Rutin standard curve, the TFC was calculated and found to be 203.12 1.65 mg/g dry weight in LSES. The TAC of LSES was expressed as ascorbic acid equivalent. The TAC of LSES was found to be 177.08 ± 3.20 mg/g dry weight. The TPACC of LSES was determined from the standard curve of catechin, and LSES was found to possess the TPACC amount of 493.75 ± 62.50 mg/g of dry weight in the context of catechin.

**TABLE 2:** Quantitative analysis of relevant antioxidants such as total flavonoid content (TFC), total phenolic content (TPC), total antioxidant capacity (TAC) and total proanthocyanidin content (TPACC) capacities of LSES

Sample name	TPC	TFC	TAC	TPACC
LSES	130.72 ± 1.09	203.12 ± 1.65	177.08 ± 3.20	493.75 ± 62.50

	Antioxidant capacities of LSES			
	IC_50_ values µg/mg			
	DPPH free radical scavenging activity	Iron‐chelating activity	Nitric oxide scavenging activity	Hydroxyl radical scavenging activity
Standard	9.22 ± 0.80 (ascorbic acid)	48.39 ± 1.87 (ascorbic acid)	3.06 ± 0.64 (quercetin)	163.87 ± 3.35 (catechin)
LSES	33.58 ± 5.49	60.61 ± 2.31	28.39 ± 2.64	326.83 ± 3.64

Note

Here, total flavonoid content (TFC), total phenolic content (TPC), total antioxidant capacity (TAC) and total proanthocyanidin content (TPACC) of LSES were expressed as mg/g of dry weight. All the IC_50_ values of LSES are expressed as (µg/ml). Values are presented as mean ± SD.

Table 2 also presents the antioxidative capacities of LSES in the form of DPPH free radical scavenging action, iron‐chelating action, nitric oxide scavenging action and hydroxyl radical scavenging action. The half‐maximal inhibitory concentration (IC_50_) to scavenge the DPPH radicals was found to be 33.58 ± 5.49 µg/ml for LSES and 9.22 ± 0.80 µg/ml for ascorbic acid, the antioxidative reference agent. The IC50 values for iron chelation were 60.61 ± 2.31 µg/ml for LSES and 48.39 ± 1.87 µg/ml for ascorbic acid, the antioxidative reference agent. In the nitric oxide scavenging assay, a dose‐dependent effect was observed, while LSES showed the IC50 value of 28.39 ± 2.64 µg/ml, which was close to that of quercetin (3.06 ± 0.64 µg/ml), the reference antioxidative agent. Finally, in the hydroxyl radical scavenging assay, a dose‐dependent reduction of hydroxyl radical was observed for LSES and standard catechin. The IC50s of LSES and catechin were respectively 326.83 ± 3.64 µg/ml and 163.87 ± 3.35 µg/ml.

#### 3.3.2 Effect of LSES on lipid peroxidation inhibition, membrane stabilization and protein denaturation inhibition

The effect of LSES on the inhibition of lipid peroxidation, membrane stabilization and inhibition of protein denaturation is summarized in Table 3. A dose‐dependent inhibition of lipid peroxidation by LSE and standard catechin was observed in this research. The IC50 value for LSES was 60.13 ± 2.39 µg/ml and 43.824 ± 3.125 µg/ml for standard catechin. Membrane stabilization activities of LSES and reference standard (ascorbic acid) in the context of IC50 values were respectively 1.92 ± 0.93 µg/ml and 1.320 ± 0.281 µg/ml. The values are statistically significant (*p* < 0.05) to each other. The inhibition of protein denaturation by LSES and reference standard are also expressed in terms of IC50, which was 161.79 ± 5.32 µg/ml for LSES and 161.79 ± 5.32 µg/ml for ascorbic acid, the reference standard.

**TABLE 3:** Lipid peroxidation activity, membrane stabilization activity and protein denaturation activity of LSES

Extract name	IC_50_ values µg/ml		
	Lipid peroxidation activity	Membrane stabilization activity	Inhibition of protein denaturation activity
Standard	43.82 ± 3.13 (Catechin)	1.32 ± 0.28 (Aspirin)	110.56 ± 5.23 (Aspirin)
LSES	60.13 ± 2.39	1.92 ± 0.93	161.79 ± 5.32

Note

Here, all the IC_50_ values are expressed as (µg/ml). Values are presented as mean ± SD.

### 3.4 Effect of LSES on the inhibition of α‐amylase

**FIGURE 1:**
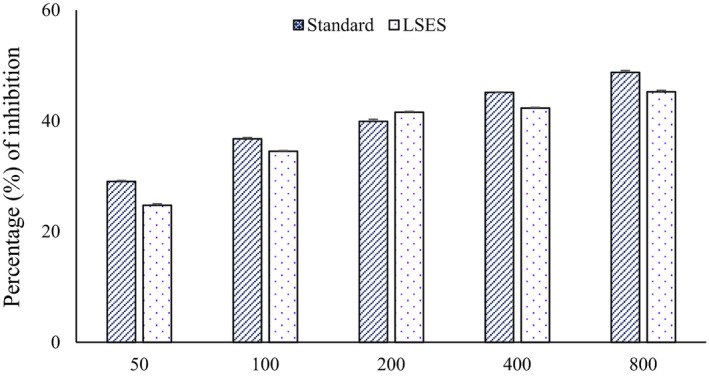
Percentage of α‐amylase inhibition of standard (Acarbose), LSES. All data were analysed using the statistical software SPSS (Statistical Package for the Social Sciences, Version 22.0; IBM Corporation, NY), followed by Tukey’s post hoc test for significance. *p* < 0.05 was considered as significant. The alphabetical notation with a‐c (*p* < 0.001 = a; *p* < 0.01 = b; and *p* < 0.05 = c) on the bar graph represents the values are significant compared with each other for the intervention period

Figure 1 shows the inhibitory effects of LSES and the reference standard acarbose on a‐amylase. The per cent of α‐amylase inhibition increases with the sample, and standard concentration increases. The α‐amylase inhibition of LSES was statistically significant (*p* < 0.05) compared with acarbose, the standard amylase inhibitory enzyme.

### 3.5 In vivo test result

#### 3.5.1 Acute toxicity evaluation

All the treated rats showed neither any toxic effect, nor any lethal effect. The present study shows administration of dose up to 2000 mg/kg did not reveal any signs of toxicity or mortality in rats during the entire observation period. Therefore, LD_50_ of LSES extract may be greater than 2000 mg/kg (2 g/kg).

#### 3.5.2 Effect of LSES on weekly body weight, blood glucose levels and oral glucose tolerance

**FIGURE 2:**
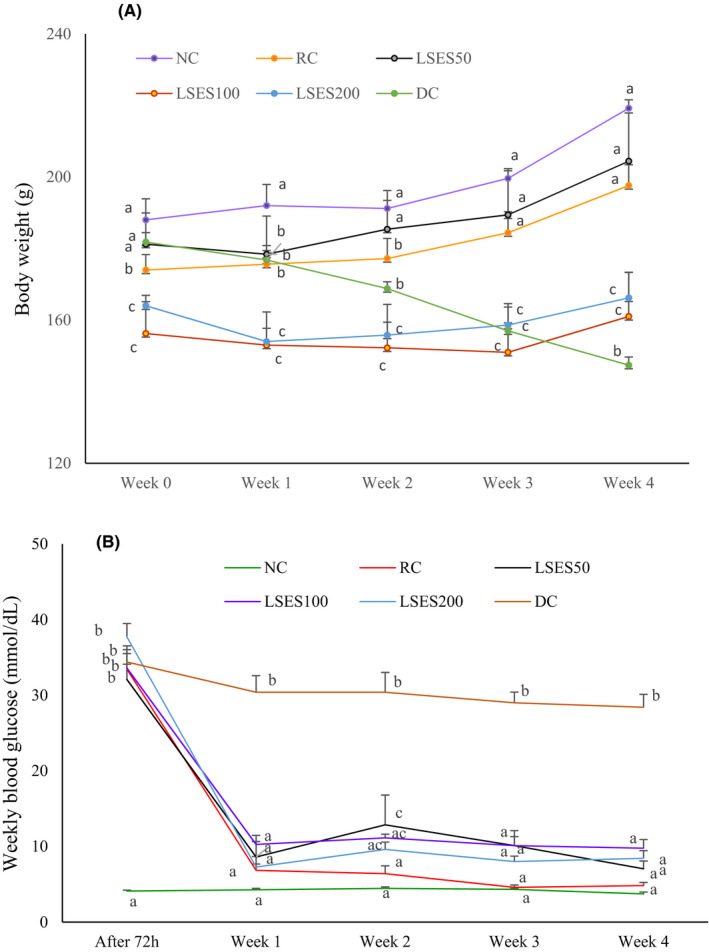
(A) Body weight changes; (B) weekly blood glucose concentration for the intervention of LSES; and (C) oral glucose tolerance test in Albino rats over four weeks at certain temperature and pressure (*n* = 6). Data are expressed as mean ± SEM. All data were analysed by the statistical software SPSS (Statistical Package for the Social Sciences, Version 22.0; IBM Corporation, NY) followed by Tukey’s post hoc test for significance. *p* < 0.05 was considered as significant

The body weight of distinct experimental animal groups during the intervention period is displayed in the graph in Figure 2A. During the intervention period, the body weights of the animals fluctuated. Except for the NC group, all groups' body weight decreased significantly (*p* < 0.05) after the first week. However, the body weights of all groups increased significantly (*p* < 0.05) after the fourth week, except for the DC group, which gradually declined significantly (*p* < 0. 05).

The animals' blood glucose levels during the intervention period are depicted in the graph below (Figure 2B). The DC group had significantly higher blood glucose levels than the other groups (*p* < 0.05). The blood glucose levels of distinct groups exhibited a substantial (*p* < 0.001) decrease in the first week of intervention. Some of them were almost the same as the NC group.

#### 3.5.3 Effect of LSES on the weights of liver, kidney, and pancreas and oral glucose tolerance

Table 4 presents the changes in the liver, kidney and pancreas weights. The liver weight of each group is significantly (*p* < 0.05) higher than the DC group except for LSES100. Conversely, each group kidney weights were significantly (*p* < 0.05) lower than the DC group. The kidney weights of the LSES50 and LSES200 groups were essentially identical to those of the NC and RC groups. However, the pancreas weights of the LSES200 group were significantly (*p* < 0.001) higher than all groups. All other treatment groups also showed higher pancreatic weights than the DC group.

**TABLE 4:** Effect of LSES on liver, kidney and pancreas weights in the intervention period

Groups	Liver weight ± SD	Kidney weight ± SD	Pancreatic weight ± SD
NC	9.32 ± 0.051^a^***	1.68 ± 0.024^a^**	0.67 ± 0.016^a^***
RC	8.53 ± 0.090^b^***	1.71 ± 0.021^a^*	0.47 ± 0.016^b^**
LSES50	9.17 ± 0.047^c^***	1.72 ± 0.015^a^*	0.68 ± 0.026^c^***
LSES100	6.14 ± 0.062^d^***	1.56 ± 0.024^b^***	0.51 ± 0.020^b^*
LSES200	7.25 ± 0.037^e^*	1.70 ± 0.041^a^**	0.56 ± 0.021^d^***
DC	6.34 ± 0.130^d^	1.81 ± 0.026^c^	0.40 ± 0.014^b^

Note

Liver, kidney and pancreas weight for the intervention of LSES in Albino rats over four weeks at certain temperature and pressure (*n* = 6). Data are expressed as mean ± SD. All data were analysed by the statistical software SPSS (Statistical Package for the Social Sciences, Version 22.0; IBM Corporation, NY) followed by Tukey’s post hoc test for significance. *p* < 0.05 was considered as significant when compared to the DC group. The star mark on the data represents the values are significant compared with each other for the intervention period. Here, ***p < 0.001; **p < 0.01; and *p < 0.05.

The superscript letters (a‐e) denote the level of significance of the experimental groups at least in the experimental environment.

After the 4^th^ week of intervention, blood glucose levels of all groups were less than 16.66 mmol/L except for the DC group. The oral glucose tolerance test (OGTT) data were collected during the third week of the experiment (Table 5). The DC group's glucose tolerance was considerably (*p* < 0.001) lower than the other groups. The glucose tolerance ability of the LSES50 group was significantly (*p* < 0.001) higher than that of the other groups, and it was comparable to that of the RC group.

**TABLE 5:** Effect of LSES on the oral glucose tolerance in the experimental animal model

Group	Blood glucose level (mmol/dl)				
	Pretreatment	Post‐treatment			
	0 min	30 min	60 min	90 min	120 min
NC	3.63 ± 0.23^a^	4.50 ± 0.88^a^	3.75 ± 0.54^a^	3.70 ± 0.11^a^	4.08 ± 0.08^a^
RC	4.75 ± 0.41^a^	14.53 ± 2.30^ab^	8.35 ± 1.04^a^	7.35 ± 2.06^a^	4.95 ± 0.51^a^
DC	28.1 ± 0.69^b^	34.06 ± 1.18^c^	27.00 ± 1.14^b^	26.40 ± 0.93^b^	27.00 ± 1.41^b^
LSES50	10.33 ± 3.82^a^	16.80 ± 2.88^b^	16.20 ± 4.03^ac^	12.98 ± 3.06^ac^	8.15 ± 1.55^a^
LSES100	23.25 ± 5.24^b^	26.28 ± 4.81^bc^	21.25 ± 4.34^bc^	15.90 ± 4.54^c^	8.15 ± 5.57^a^
LSES200	27.10 ± 1.23^b^	21.98 ± 1.96^b^	19.23 ± 1.60^bc^	17.23 ± 1.30 ^c^	14.70 ± 0.89^a^

Note

Oral glucose tolerance capacity of experimental animals. Data are presented as mean ± SD. All data were analysed by the statistical software SPSS (Statistical Package for the Social Sciences, Version 22.0; IBM Corporation, NY) followed by Tukey’s post hoc test for significance. *p* < 0.05 was considered as significant. The superscript letters a–c denote the level of significance of experimental groups at least in the experimental conditions.

### 3.6 Effect LSES on liver glycogen and other biochemical parameters

Table 6 summarizes the effects of LSES on animal liver glycogen, serum alanine aminotransferase (ALT), serum aspartate aminotransferase (AST), lipid profiles, lactate dehydrogenase (LDH), creatinine kinase‐MB (CK‐MB), urea, creatinine and uric acid levels. The concentrations of liver glycogen in the treatment groups, RC and NC, were considerably (*p* < 0.001) lower than in the DC group.

**TABLE 6:** Effects of LSES on different serum parameters and liver glycogen

Parameters	NC	RC	LSES50	LSES100	LSES200	DC
Urea (mg/dl)	47.0 ± 1.2^a^*	45.2 ± 1.3^b^**	43.0 ± 1.58^c^***	38.6 ± 1.21^d^*	48.8 ± 0.80^a^***	56.2 ± 2.70^e^
Uric acid (mg/dl)	6.17 ± 0.05^a^**	5.88 ± 0.13^b^***	5.09 ± 0.04^c^***	5.50 ± 0.04^c^***	5.67 ± 0.03^b^**	6.46 ± 0.03^d^
Creatinine (mg/dl)	0.52 ± 0.02^a^***	0.55 ± 0.02^b^***	0.58 ± 0.02^c^**	0.52 ± 0.3^abc^***	0.61 ± 0.01^d^***	0.90 ± 0.02^e^
Liver glycogen (mg/g)	0.254 ± 0.001^a^***	0.201 ± 0.007^b^**	0.164 ± 0.002^c^***	0.039 ± 0.001^d^***	0.085 ± 0.001^e^***	0.414 ± 0.011^f^
Total cholesterol (mg/dl)	62.4 ± 2.11^a^***	69.6 ± 1.08^b^*	58.4 ± 1.89^c^***	62.0 ± 2.35^a^***	61.4 ± 2.59^a^***	76.0 ± 0.70^d^
TG (mg/dl)	111.0 ± 4.14^a^***	42.2 ± 1.88^b^***	80.0 ± 1.52^c^***	128.4 ± 3.36^d^***	120.2 ± 2.39^e^***	167.8 ± 2.96^f^
LDL (mg/dl)	47.6 ± 1.47^a^**	37.6 ± 1.03^b^***	41.6 ± 1.51^c^***	45.4 ± 1.63^d^*	42.4 ± 1.29^c^***	52.0 ± 1.02^e^
HDL (mg/dl)	13.8 ± 0.37^a^*	16.6 ± 0.50^b^***	13.2 ± 0.37^c^*	13.8 ± 0.38^a^**	14.6 ± 0.51^d^**	11.6 ± 0.51^e^
AST (U/L)	75.2 ± 1.85^a^**	85.3 ± 1.44^b^***	125.6 ± 2.11^c^***	153.4 ± 7.01^d^***	97.0 ± 7.87^e^***	170.0 ± 2.24^f^
ALT (U/L)	73.5 ± 1.36^a^***	48.3 ± 1.66^b^***	81.0 ± 1.23^c^***	91.0 ± 1.28^d^***	67.2 ± 2.18^e^***	97.3 ± 1.86^f^
LDH (U/L)	547.6 ± 5.23^a^***	539.0 ± 6.41^b^***	580.0 ± 4.69^c^***	741.0 ± 6.68^d^***	536.0 ± 5.55^a^***	782.6 ± 5.09^e^
CK‐MB (U/L)	171.2 ± 4.12^a^***	87.6 ± 3.91^b^***	103.4 ± 7.81^c^***	126.6 ± 1.92^d^***	118.2 ± 2.58^e^***	220.8 ± 2.42^f^

Note

Serum urea, uric acid, creatinine, liver glycogen, total cholesterol, TG, LDL, HDL, AST, ALT, LDH and CK‐MB for intervention of LSES in Albino rats over four weeks at certain temperature and pressure (*n* = 6). Data are expressed as mean ± SD. All data were analysed by the statistical software SPSS (Statistical Package for the Social Sciences, Version 22.0; IBM Corporation, NY) followed by Tukey’s post hoc test for significance. *p* < 0.05 was considered as significant when compared to the DC group. The star mark on the data represents the values are significant compared with each other for the intervention period. Here, ***p < 0.001; **p < 0.01; and *p < 0.05.

The superscript letters (a‐f) denote the level of significance of the experimental groups at least in the experimental environment.

The treatment groups' serum ALT levels were considerably (*p* < 0.001) lower than the DC group. LSES200 was found to be the most effective dose for lowering ALT levels compared with other doses. The treatment groups' serum AST levels were considerably (*p* < 0.001) lower than the DC. LSES200 showed the highest efficacy in managing the AST levels of STZ‐induced animals. Table 6 demonstrates that the cholesterol levels of the treatment groups were considerably (*p* < 0.001) lower than the DC group in terms of total cholesterol, triglyceride (TG), low‐density lipoprotein (LDL) and high‐density lipoprotein (HDL). LSES50 was found to significantly (*p* < 0.001) reduce the total cholesterol compared with other groups. LSES50 has lower triglyceride when compared to other groups except for the RC group. The treatment groups' HDL levels were considerably greater (*p* < 0.05) than the DC group, while LSES200 showed higher HDL than the NC group. Treatment groups' LDL levels were found to be considerably (*p* < 0.05) lower than the DC group, with LSES50 having lower LDL than all other groups except for the RC group.

The DC group showed increased serum LDH and CK‐MB levels compared with the NC, RC and treatment groups. LSES200 was highly effective in reducing LDH levels than any other group except for the RC group; on the contrary, LSES50 was highly effective in reducing CK‐MB levels than any other groups except for the RC group.

Serum urea, creatinine and uric acid values are decreased significantly (*p* < 0.05) compared with the DC group. LSES100 shows a lower serum urea and creatinine level than any other treatment group near the NC and RC groups. On the contrary, LSES50 showed lower serum uric acid when compared to other groups.

#### 3.6.1 Histopathological analyses of pancreas and kidneys

**FIGURE 3:**
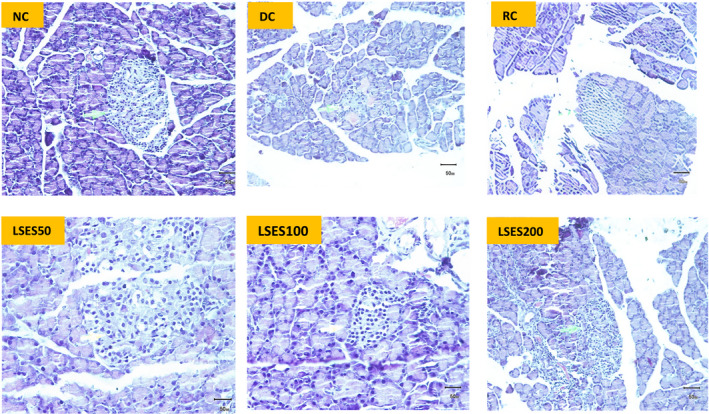
Histopathological image (H & E staining × 125) of pancreatic tissue of the experimental animals from different groups. The arrow shows the pancreatic islet of Langerhans (microscopic resolution: 10 × 40). Micrographs of haematoxylin and eosin staining of rat pancreas. Light microscopies of pancreatic sections stained with PAS and counterstained with haematoxylin are shown

**FIGURE 4:**
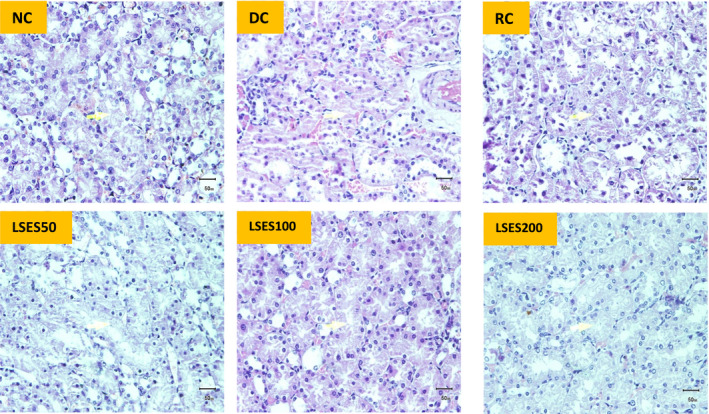
Histopathological image of kidney tissue of the experimental animals from different groups. The arrow shows the glomerulus of kidney cells (microscopic resolution: 10 × 40). Micrographs of haematoxylin and eosin staining of rat kidney. Light microscopies of glomerulus sections stained with PAS and counterstained with haematoxylin are shown

The experimental parameters' histopathological findings were scored as follows: (−) denotes ‘no abnormality’, with an injury extent of less than 5%, (+) shows ‘mild injury’, with an injury extent of 5–25 per cent, (++) suggests ‘moderate injury’, with an injury extent of 25–50 per cent, and (+++) indicates ‘severe injury’. The histopathological images and scoring for pancreatic and kidney tissues of different experimental animal groups are shown in Figures 3 and 4 and Table 7. Compared with NC, the size of the islet of Langerhans in the pancreas was shown to be smaller in the DC group and degeneration. On the contrary, other treatment groups showed less degeneration, with the width of the islet of Langerhans and the area occupied by β‐cell/islet of Langerhans being much higher than the DC group. In addition, the kidney tissues of the DC and RC groups show more tubular epithelial cell degeneration, tubular epithelial cell necrosis and hyperaemic interstitial arteries than the LSES groups, whereas LSES100 was shown to be more promising than the other two doses.

**TABLE 7:** Changes in histopathological morphology of pancreatic cells

	NC	DC	RC	LSES50	LSES100	LSES200
Changes in pancreatic tissues						
Degenerated cells	−	+++	+			
Necrotic cells	−	+++	+			
Diameter of islet of Langerhans (μm)	173 ± 47	ND	125 ± 28	325 ± 28.86	140 ± 23.09	145 ± 51.96
Area occupied by β‐cell/islet of ± Langerhans (μm^2^)	20,703 ± 4730	ND	11,227 ± 2309	94,500 ± 3660	17,920 ± 1920	19,000 ± 1450
Changes in kidney tissues						
Atrophic glomerulus and tubules	−	++	−	−	−	+
Eosinophilic secretion in the tubule’s lumen	−	+	−	−	−	+
Hyperaemic vessels in the interstitium	−	+	−	−	+	+
Increased fibrous tissue	−	+	−	−	−	+
Interstitial mononuclear cell titration	−	+	+	+	+	+
Tubular epithelial cell degeneration	−	+	−	−	−	−
Tubular epithelial cell necrosis	−	+	−	−	−	+

Note

Histopathological assessments of the experimental parameters are graded as follows: (–) indicates ‘no abnormality’; (+) indicates ‘mild injury’; (+ +) indicates ‘moderate injury’; (+ + +) indicates ‘severe injury’

### 3.7 Computational studies

#### 3.7.1 Molecular docking (LSES)

**FIGURE 5:**
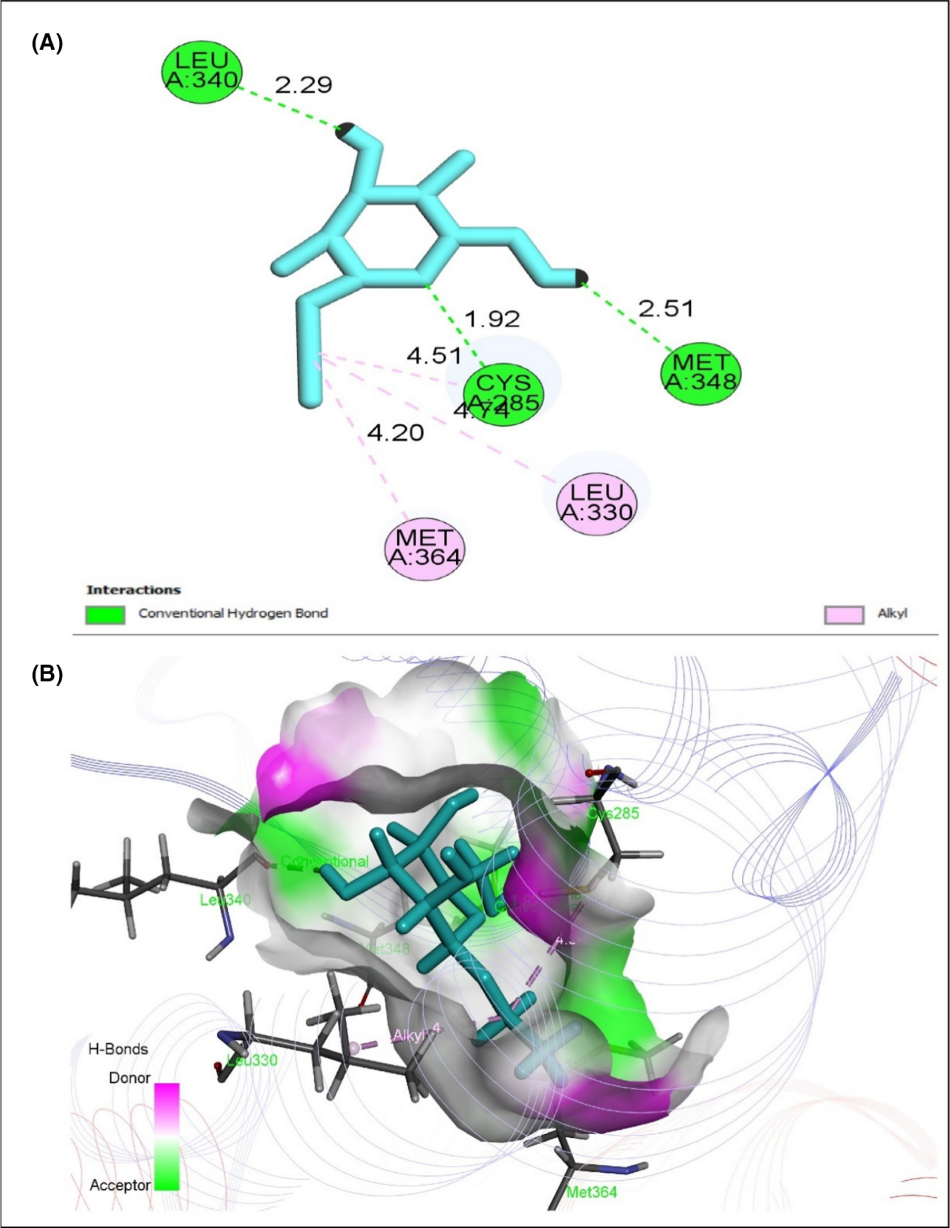
Best rank poses of (A) 2D and (B) 3D molecular interactions of ethyl alpha‐d‐glucopyranoside docked with the active‐site PPAR‐γ for antidiabetic potential

**FIGURE 6:**
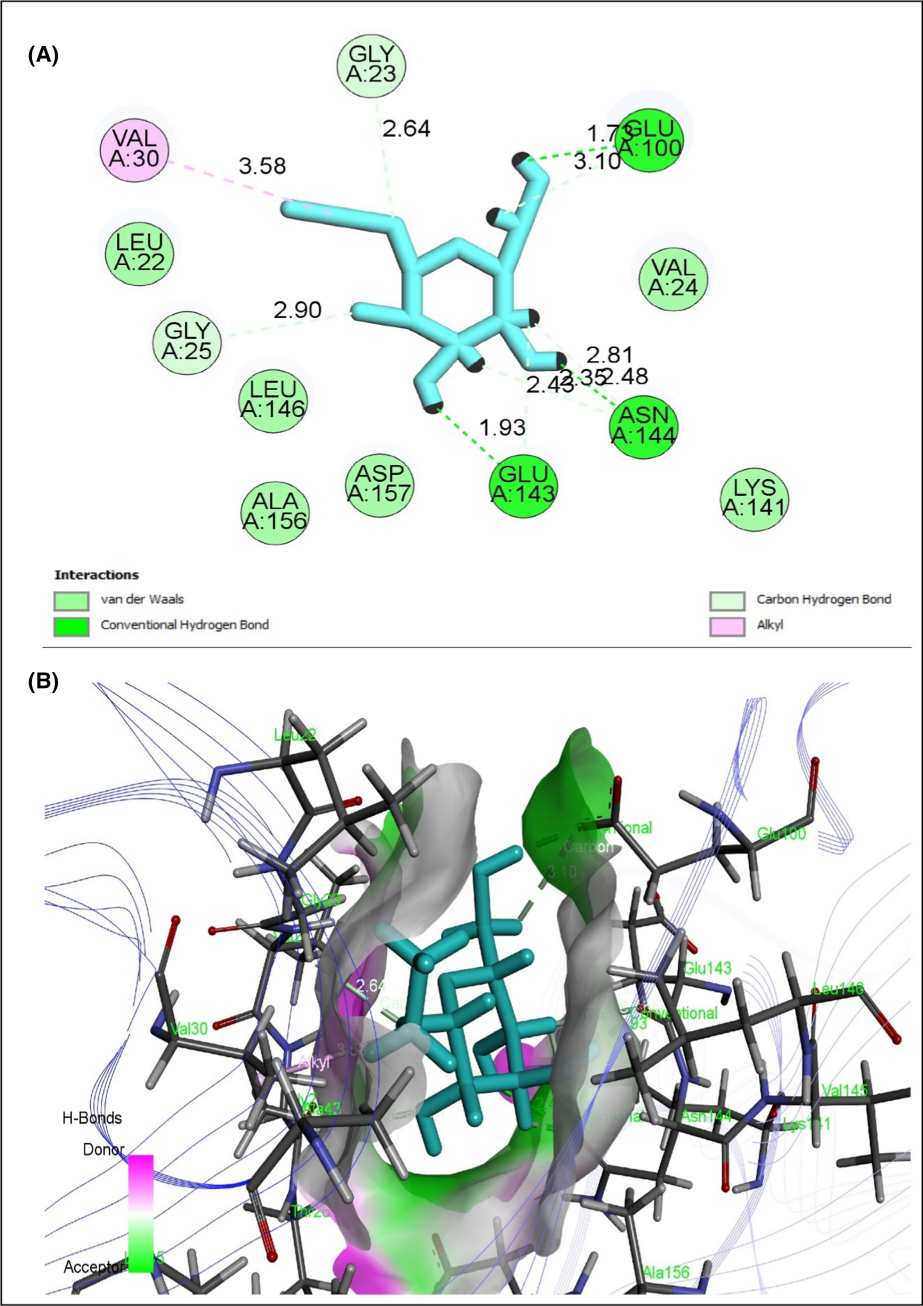
Best rank poses of (A) 2D and (B) 3D molecular interactions of ethyl alpha‐d‐glucopyranoside docked with the active‐site AMPK for antidiabetic potential

**FIGURE 7:**
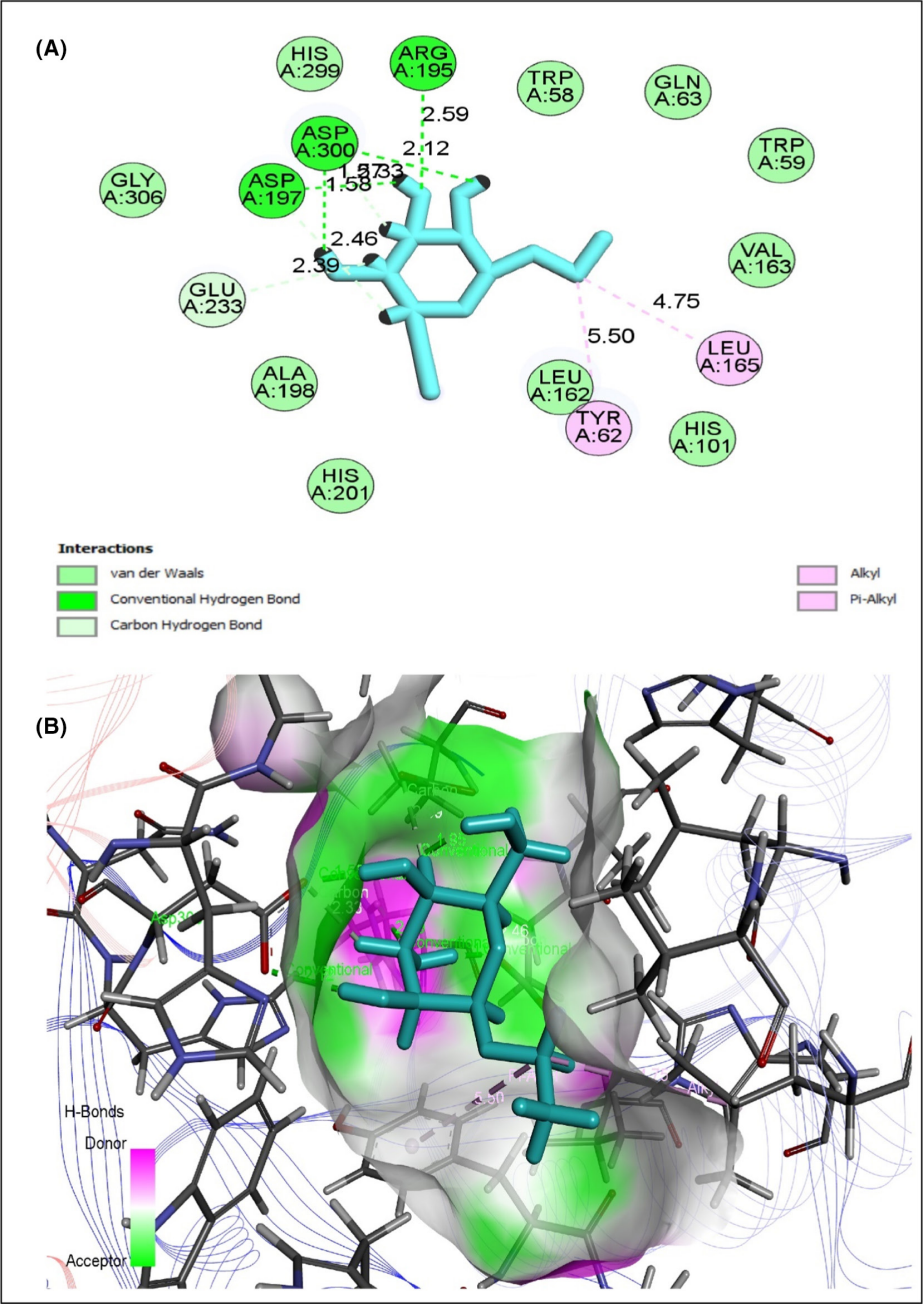
Best rank poses of (A) 2D and (B) 3D molecular interactions of ethyl alpha‐d‐glucopyranoside docked with the active‐site 1PPI α‐amylase for antidiabetic potential

To evaluate antidiabetic activity, eight selected compounds interacted against the peroxisome proliferator‐activated receptor‐gamma (PPAR‐γ, PDB ID 3G9E), AMP‐activated protein kinase (AMPK, PDB ID: 4CFH) and α‐amylase enzyme (PDB ID: 1PPI) (Table 8). All compounds exhibited an excellent binding affinity with a higher docking score (six compounds) than reference drug metformin. However, based on abundance and pharmacokinetic properties, ethyl alpha‐d‐glucopyranoside was chosen as the best candidate, which exerted the highest binding affinity against PPAR‐γ (Figure 5), AMPK (Figure 6) and alpha‐amylase (Figure 7) with docking scores −5.212 Kcal/mol, −5.236 Kcal/mol and −6.008 Kcal/mol, respectively (Table S2). Taken together all of these, the compound exerted an antidiabetic effect by acting on PPAR‐γ, AMPK and α‐amylase.

**TABLE 8:** Physiochemical properties of the selected compounds in LSES

Compounds	Lipinski’s rules				Lipinski’s violations	Veber’s rules	
	MW (g/mol)	HBA	HBD	Log P		nRB	TPSA Å
Ethyl alpha‐d‐glucopyranoside	208.2	6	4	−2.07	0	3	99.3
Beta‐D‐Ribopyranoside, methyl 2,3,4‐tri‐O‐methyl‐Ribopyranoside	206.24	5	0	−0.94	0	4	46.15
Hexanoic acid, octadecyl ester	368.6	2	0	6.0	1	22	26.30
Hexadecanoic acid, 2‐hydroxy‐1‐(hydroxymethyl) ethyl ester	330.50	4	2	3.18	0	18	66.76
Octadecanoic acid, 2,3‐dihydroxypropyl ester	358.56	4	2	3.63	0	20	66.76
13‐Docosenamide, (Z)‐	337.58	1	1	5.06	1	19	43.09
Acetoxyacetic acid, 2‐(1‐adamantyl) ethyl ester	280.36	4	0	2.77	0	7	52.60
Phenol, 4‐(1,1,3,3‐tetramethylbutyl)‐	206.32	1	1	3.87	0	3	20.23

Abbreviations: HBA, hydrogen bond acceptor (≤10); HBD, hydrogen bond donor (≤5); Log P, lipophilicity (≤4.15); MW, molecular weight (≤500 g/mol); nRB, number of the rotatable bond (≤10); TPSA, topological polar surface area (≤140).

#### 3.7.2 Pharmacokinetic properties (LSES)

Using Lipinski's rule of five and Veber's rules (absorption, distribution, metabolism and excretion/transport), the researchers discovered the drug‐like features of selected substances. Only two compounds defied one of Lipinski's five principles, while the others followed both Lipinski's and Veber's standards (Table 8). As a result, all compounds revealed drug‐like properties and could be taken orally.

## 4 DISCUSSION

Diabetes mellitus is a major public health issue that affects people all over the world. Cardiovascular disease, neuropathy, nephropathy and retinopathy are all severe complications of diabetes. Diet, physical activity and current medications can all help in the control of diabetes (insulin or oral hypoglycaemic drugs such as sulfonylureas and biguanides). Different extracts from medicinal plants have been used to treat diabetes in the past in various parts of the world. In this study, we investigated the antidiabetic activity of *Lasia spinosa* (ethanolic extract) or LSES. In this study, streptozotocin (STZ) was utilized to produce diabetes mellitus in experimental rats. It produces pancreas swelling in adult Wister rats, eventually causing degeneration in Langerhans islet beta cells, and inducing experimental diabetes mellitus in 2–4 days.^9^ Diabetic rats are compared with normal rats and LSES‐treated rats in this study.

The alkaloids, flavonoids, steroids, tannins, carbohydrates and protein are found in phytochemical screening. Flavonoid, tannin and alkaloid are some of the chemicals derived from the plants that have been shown to lower blood glucose levels.^31^ Thus, the strong antidiabetic impact of LSES could be attributed to the presence of these components in the extracts, which could act synergistically or independently to boost glycolytic enzyme activity. In addition, some flavonoids have been shown to have antidiabetic properties. For example, quercetin can improve hyperglycaemia, hypertriglyceridaemia and hypercholesterolaemia in STZ‐diabetic rats.^32^ Furthermore, considerable flavonoid content was found in LSES, which may reduce blood glucose levels in rats.

Polyphenols are effective in the treatment of a variety of disorders because of their antioxidant, anti‐inflammation, antifibrotic and metabolic regulatory properties, and one of the most intensively investigated biological activities of polyphenols is antidiabetic activity.^33^ The presence of TPC in LSES may help ameliorate the complications related to diabetes. Long‐term difficulties of diabetes mellitus are linked to oxidative reactions, increased free radical production and oxidative stress, all of which contribute to diabetic tissue damage.^34^ Antioxidants help to damage these free radicals and help to reduce oxidative stress. Total antioxidant capacity (TAC) and total proanthocyanidin contents (TPACC) are present in LSES, which may help to regulate this free radical and improve diabetes complications. By activating NOSs and NADPH oxidase, hyperglycaemia also causes an excess of NO* and O* to be generated. O2* reacts with NO* to generate OONO*, an oxidant that causes tissue injury, as well as lipid, protein and nucleic acid damage, resulting in oxidative stress and endothelial dysfunction. In oxidative stress‐related disorders, hydroxyl radicals (OH) produced in the human body may play a key role in tissue injury at inflammatory locations. Free radicals (Fe2+« Fe3+) are generated by transition elements (e.g. iron), and iron (II) ions are known to cause ascorbic acid and glucose oxidation.^34^ Lipid peroxidation is also increased in diabetes, which could be related to the increased generation or decreased elimination of reactive oxygen species (ROS).^35^ LSES extract has DPPH free radical scavenging activity, iron‐chelating activity, nitric oxide scavenging activity, hydroxyl radical scavenging activity, lipid peroxidation activity, protein denaturation inhibition activity and membrane stabilization activity.

Inhibitors of α‐amylase slow the breakdown of carbohydrates and reduce the postprandial blood glucose spike in people with diabetes.^36^ Controlling the catalytic activity of this enzyme lowers glucose production in the postprandial stage, which could be a therapeutic advantage for people with diabetes. The α‐amylase inhibitory activity of LSES will be a game‐changer for diabetic rats for the management of diabetic complications. During the experiment, it was revealed that the DC group's body weight was severely reduced. The treatment group's body weight dropped as well, although it was restored after a few days; NC has no decrement. This decrement was attributed to muscle and adipose tissue loss caused by an increase in tissue protein and fatty acid breakdown driven by a fall in plasma insulin levels. Protein synthesis is hindered, and breakdown is increased in the absence of insulin, resulting in an increase in amino acid levels in the blood, which can subsequently be used for gluconeogenesis.^37^

When diabetic rats were compared to normal control rats, their fasting blood glucose levels were considerably higher. LSES treatment lowered fasting blood glucose levels considerably, and LSES50 has higher activity than the RC group. The extract's antidiabetic properties could be linked to secondary metabolites found in the plant. The glucose tolerance test (OGTT) assesses the body's ability to metabolize and remove sugar (glucose) from the bloodstream. The OGTT can be used to detect diabetes 1, gestational diabetes (diabetes during pregnancy) and prediabetes, among other things (high blood sugar that predicts type 2 diabetes). All treatment groups in this experiment showed better glucose tolerance than the diabetic control group. These results could be owing to the STZ‐induced diabetic rats receiving LSES, which caused the pancreas to heal and secrete insulin into the bloodstream.

In the current investigation, STZ‐treated DC rats had an increase in kidney weight in proportion to their body weight when compared to other groups. The overexpression of transforming growth factor (TGF)‐beta 1 in the kidney, particularly in proximal convoluted tubule (PCT) cells and glomerular mesangial cells, has been linked to the development of renal hypertrophy in insulin‐dependent diabetic mellitus (IDDM). The observed data of pancreatic weight of the DC group were lower than other groups. The disruption and loss of pancreatic islets, as well as selective killing of insulin‐producing cells, may account for the DC group's decreased pancreas weight.^38^ The liver weight of the treatment group was higher than the DC group, and it may be a detrimental consequence of diabetes. Even though almost all the liver glycogen in the treatment groups was lost after 24 h of fasting, STZ‐induced diabetic rats preserved a large amount of hepatic glycogen. The blood glucose level influences the amount of residual liver glycogen. The current findings show that hyperglycaemia may be to blame for the delayed clearance of liver glycogen in fasting STZ‐diabetic rats.^39^

ALT is a cytoplasmic enzyme found in abundance in the liver; an increase in ALT in the blood indicates liver disease; however, AST is less specific as a marker of liver injury. Hepatocellular injury may be the reason for the high levels of AST and ALT in the serum of STZ‐induced diabetic rats.^40^ In the present investigation, ALT and AST levels are higher in the DC group when compared to other groups. The lower levels of ALT and AST in the treatment groups indicate that LSES has a hepatoprotective effect.

Fatty liver, hypercholesterolaemia and hypertriglyceridaemia are all complications of diabetes mellitus.^41^ Furthermore, high cholesterol levels are also linked to diabetic nephropathy. In the present research, the serum lipid levels (cholesterol, TG and LDL) of the NC, RC and LSES‐treated diabetic rats were significantly lower than DC group rats. On the contrary, HDL levels decreased in the DC group compared with other groups, and several investigations suggested that HDL levels decrease in diabetic patients. The ability of the extract to maintain lipid profiles in extract‐treated rats demonstrates its efficacy against experimental type 2 diabetic rats.^42^

In the case of diabetic cardiomyopathy, a diagnosis of cardiac enzymes is required. Biomarkers for myocardial infarction include aspartate aminotransferase (AST), creatine kinase‐isoenzyme (CK‐MB) and lactate dehydrogenase (LDH). These enzymes are closely bound to the contractile apparatus of cardiac muscle tissue, and they are released into the bloodstream when the heart muscle is injured. In diabetic animals, blood levels of CK‐MB and LDH are increased.^43^ However, compared with the diabetic control (DC) group, LSES dramatically reduced levels of cardiac enzymes such as CK‐MB, AST and LDH, showing that it prevents heart damage and offers cardioprotection.

In this investigation, plasma urea, uric acid and creatinine levels in experimental rats were measured. These values are thought to be important indicators of renal impairment, and studies show that elevated plasma creatinine and urea levels in diabetic patients may suggest a prerenal issue^43^ and renal impairment is a common complication for diabetic patients. The present study showed that extract of *Lasia spinosa* is capable of lowering kidney function parameters when compared to the DC group. In addition, the result showed that the extract of *Lasia spinosa* has the potential to be nephron‐protective.

Control animals' pancreas histopathology revealed normal pancreatic parenchyma cells and islet cells. The pancreas segment of diabetic controls exhibited islet cell necrosis, oedema, inflammation, and alterations in the diameter of the islet of Langerhans and the area occupied by β‐cell/islet of Langerhans. The RC group showed high recovery of damaged cells, and LSES100 LSES200 showed significant recovery compared with the diabetic control group. Histopathological examination of kidney sections from STZ‐induced diabetic rats (DC group) revealed severe tubular vacuolar degeneration, increased glomerular space and a variety of other abnormalities, including hyperaemic vessels in the interstitium, increased fibrous tissue, interstitial mononuclear cell titration, tubular epithelial cell degeneration and tubular epithelial cell necrosis. On the contrary, no changes were observed in the NC group, and the RC group's recovery rate is almost the same as the NC group. The changes mentioned above were dramatically reduced after treatment with LSES, showing a preventive role in renal injury. The LSES100 treatment group recovered faster than the other treatment groups.

In structural molecular biology and computer‐assisted drug design (CADD), a molecular docking study is vital for developing a new drug, and a molecular docking tool evaluates the prediction of new compound binding interactions against key proteins.^44^ Molecular docking is also utilized to find out what the putative molecular mechanisms of action of specific pharmacological agents are.^45^ However, molecular docking was used to understand further the molecular mechanism and its relationship to the study's findings. A total of eight compounds were chosen by PASS prediction from LSES GC‐MS data to understand the biological activity better. These chemicals were tested against the active sites of the peroxisome proliferator‐activated receptor‐gamma (PPAR, PDB ID: 3G9E), AMP‐activated protein kinase (AMPK, PDB ID: 4CFH) and the α‐amylase enzyme (PDB ID: 1PPI). Some compounds exhibited higher binding affinity than the widely antidiabetic drug metformin. The peroxisome proliferator‐activated receptor (PPAR) stimulates some genes in tissues, resulting in increased glucose and lipid absorption, lower free fatty acid content and reduced insulin resistance.^46^ Metformin, a common treatment for type 2 diabetes, has been shown to activate the AMP‐activated protein kinase (AMPK) in intact cells and in vivo. Inhibition of human pancreatic amylase (HPA) in the small intestine is significant in the treatment of type 2 diabetes since HPA is linked to an increase in postprandial glucose levels.^47^ This study found that when diabetic rats were given the title compounds, their blood glucose levels dropped. The title compounds may exert their hypoglycaemic effect by acting as a potent agonist or antagonist on the PPAR receptor, AMPK receptors or HPA receptor, thereby increasing the body's insulin sensitivity.

The pharmacokinetic features of several LSES phytocompounds can be represented using Lipinski's rule of five, which states that orally administered drugs should have a molecular weight of 500 amu, ten hydrogen bond acceptor sites, five hydrogen bond donor sites and a lipophilicity value of Log P 4.15, as well as Veber's rule of two (ten rotatable bonds, topological polar surface area of 140). If medicine or chemical breaks all these requirements, it is not regarded to have good oral bioavailability.^48^ However, this study found that some compounds in LSES have a high oral bioavailability of the bioactive substances tested. As a result, based on a study depicted as a drug‐like quality, these phytocompounds could be viewed as viable medication candidates with good oral bioavailability. It is obvious from the preceding discussions that LSES is very efficient in treating type 2 diabetes because it reduces the diabetes‐related parameters in the STZ‐induced rat model; histological examination and computational studies have proven its relevance.

## 5 CONCLUSION

The injection of LSES to STZ‐diabetic rats decreased weekly blood glucose levels, normalized lipid profiles, improved liver and cardiac indicators and increased glucose tolerance ability in the current study. Most importantly, LSES exhibits a protective action in STZ‐induced diabetic pancreas and kidneys. In in vitro studies, LSES extract showed antioxidant potentiality and anti‐inflammatory and antidiabetic activity. Furthermore, the antidiabetic activity of compounds from GC‐MS is substantially influenced by in silico pass prediction. More research is needed to fully understand the mechanism of LSES activity at the cellular and molecular levels.

## CONFLICT OF INTEREST

The authors declare that they have no conflict of interest.

## AUTHOR CONTRIBUTIONS

**Md. Atiar Rahman:** Conceptualization (equal); Funding acquisition (equal); Project administration (equal); Resources (equal); Supervision (equal); Validation (equal); Writing – review & editing (equal). **Md. Mamunur Rashid:** Data curation (equal); Formal analysis (equal); Investigation (equal); Methodology (equal); Writing – original draft (equal). **Md. Shahidul Islam:** Formal analysis (equal); Investigation (equal); Visualization (equal). **Md. Amjad Hossen:** Software (equal); Visualization (equal). **A. S. M. Ali Reza:** Data curation (equal); Methodology (equal); Resources (equal). **A. M. Abu Ahmed:** Data curation (equal); Supervision (equal); Writing – review & editing (equal). **Afnan M. Alnajeebi:** Funding acquisition (equal); Writing – review & editing (equal). **Nouf Abubakr Babteen:** Funding acquisition (equal); Visualization (equal); Writing – review & editing (equal). **Mala Khan:** Data curation (equal); Formal analysis (equal). **Salama Mostafa Aboelenin:** Funding acquisition (equal); Writing – review & editing (equal). **Mohamed Mohamed Soliman:** Funding acquisition (equal); Validation (equal); Writing – review & editing (equal). **Alaa H. Habib:** Funding acquisition (equal); Resources (equal). **Hend Faisal H. Alharbi Alharbi:** Funding acquisition (equal); Writing – review & editing (equal).

## Supporting information

Supplementary Material
