# Supplementary material for: Incredible affinity of Kattosh with PPAR‐γ receptors attenuates STZ‐induced pancreas and kidney lesions evidenced in chemicobiological interactions
Source: J Cell Mol Med. 2022 May 2;26(12):3343–63. doi: 10.1111/jcmm.17339 (PMC9189352; doi:10.1111/jcmm.17339)
Supplement: Supplementary file 1 — Supplementary Material [file JCMM-26-3343-s001.docx]

**Table S1. Phytochemical group tests of LSES**

| Phytochemical constituents | Test name | Observations of LSES extract |
| --- | --- | --- |
| Carbohydrates | Molisch’s test | ^+^ |
|  | Benedict test | ^+^ |
| Alkaloids | Mayer’s test | ^+^ |
|  | Wagner’s test | ^+^ |
|  | Dragendorff’s test | ^+^ |
| Cardiac glycosides | Keller-Killani test | _ |
| Flavonoids | Alkali test  Lead (II) acetate | +  + |
| Saponin | Frothing test | ^_^ |
| Steroids | Salkowski’s test  Liberman test | +  + |
| Tannins | General Color test | + |
| Rotein | Biruet’s test  Ninhydrin Test | +  + |
| Phlobatannins | General color test | _ |

**(+) Presence, (-) Absence**

**Table S2. Docking score of major bioactive compounds from GC-MS data of LSES.**

| **Compounds** | **PubChem ID** | **3G9E** | **4CFH** | **1PPI** |
| --- | --- | --- | --- | --- |
| Ethyl alpha-d-glucopyranoside | 9815668 | -5.212 | -5.236 | -6.008 |
| Hexadecanoic acid, 2-hydroxy-1-(hydroxymethyl) ethyl ester | 123409 | -5.562 | -4.791 | -3.766 |
| Hexanoic acid, octadecyl ester | 575856 | -5.307 | -4.031 | -2.925 |
| Octadecanoic acid, 2,3-dihydroxypropyl ester | 24699 | -5.091 | -5.959 | -3.127 |
| Beta-D-Ribopyranoside, methyl 2,3,4-tri-O-methyl-Ribopyranoside | 21140439 | -5.662 | -4.871 | -3.844 |
| Phenol, 4-(1,1,3,3-tetramethylbutyl)- | 8814 | -6.12 | -6.401 | -5.558 |
| 13-Docosenamide, (Z)- | 5365371 | -6.785 | -5.451 | -3.726 |
| Acetoxyacetic acid, 2-(1-adamantyl) ethyl ester | 533021 | -3.973 | -4.269 | -4.201 |
| Metformin | 4091 | -4.15 | -3.382 | -2.99 |

PPARγ (PDB ID 3G9E); AMPK (PDB ID: 4CFH) and α-amylase enzyme (PDB ID: 1PPI); Docking scores in kcal/mol; bold text indicates the highest score.


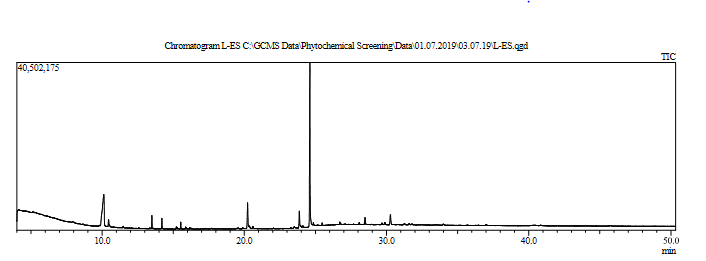


**Figure S1.** Gas chromatography-mass spectrometry proﬁle of LSES was obtained from GC-MS with electron impact ionization (EI) method on a gas chromatograph (GC17A, Shimadzu Corporation, Kyoto, Japan) coupled to a mass spectrometer (GC-MS TQ 8040, Shimadzu Corporation, Kyoto, Japan). The inlet temperature was set at 260°C and the oven temperature was programmed as 70°C (0 min); 10°C, 150°C (5 min); 12°C, 200°C (15 min); 12°C, 220°C (5 min).
